# FSD-YOLO: A Fusion Framework for Region Segmentation and Deformable Object Detection in Container Yards

**DOI:** 10.3390/s26072029

**Published:** 2026-03-24

**Authors:** Linghao Dai, Zhihong Liang, Qi Feng, Shihuan Xie, Hongxu Li

**Affiliations:** College of Big Data and Intelligent Engineering, Southwest Forestry University, Kunming 650224, China; dailinghao@swfu.edu.cn (L.D.); fengqi.fq@outlook.com (Q.F.); xieshihuan@swfu.edu.cn (S.X.); 13085315910@163.com (H.L.)

**Keywords:** safety monitoring, object detection, YOLOv8, SegFormer, deformable convolution, dynamic loss weighting

## Abstract

Safety monitoring in container hoisting operations within rail-road intermodal logistics parks is a critical task in industrial safety management. Such scenarios are characterized by complex environments, large variations in target scales, deformable object shapes, and frequent occlusions, which pose significant challenges to visual perception systems. Conventional single-task models suffer from inherent limitations in handling low recall rates for distant small targets and insufficient adaptability to geometric deformations, making them inadequate for high-precision, real-time safety warning applications. To address these challenges, this study proposes a unified visual analysis framework that integrates semantic segmentation and object detection to enhance the recognition performance of small and deformable targets in complex operational environments, enabling real-time perception and safety warning of key objects and hazardous regions within container yards. Specifically, we introduce FSD-YOLO, a fusion-based architecture composed of the following key components. First, a SegFormer-based semantic segmentation module is employed to achieve pixel-level delineation of different operational regions. Second, an improved object detection network is developed based on the YOLOv8n architecture, incorporating: (1) the integration of C2f modules in the shallow layers of the backbone to enhance high-resolution feature extraction; (2) the embedding of C2fDCN modules within the detection head to improve modeling capability for deformable objects via deformable convolution; (3) the adoption of CARAFE upsampling operators to optimize multi-scale feature fusion; and (4) a dynamic loss-weighting strategy for small objects, where loss weights are adaptively adjusted according to target area to increase training emphasis on small-scale targets. Finally, a decision-level fusion strategy is applied to combine segmentation and detection outputs, enabling real-time safety judgment based on semantic rules. Experimental results on a self-constructed container yard dataset demonstrate that the proposed detection model achieves an mAP50-95 of 0.6433 and an mAP50 of 0.9565, significantly outperforming the baseline YOLOv8n model (mAP50-95: 0.5394, mAP50: 0.8435), thereby validating the effectiveness of the proposed framework.

## 1. Introduction

With the continuous growth of global trade, multimodal transportation has become an increasingly important component of modern logistics systems. As a key hub connecting railway and road transportation, rail-road intermodal logistics parks have attracted growing attention from both academia and industry with respect to operational efficiency and safety management. Kaidabettu et al. [1] systematically reviewed key decision tasks and operational methods in inland rail-road intermodal terminals, showing that the tight coupling among terminal processes, resource coordination, and yard layout creates high operational complexity, thereby imposing stringent requirements for park-level safety management and risk control.

Within these logistics parks, container yards serve as core functional areas responsible for loading and unloading, transshipment, and temporary storage, and are among the most operation-intensive zones. During yard operations, large-scale lifting equipment, transport vehicles, and on-site personnel frequently operate in close proximity within limited spaces, resulting in safety risks that exhibit strong spatial correlations and dynamic variations. Wu et al. [2] investigated crane operation scenarios and pointed out that unauthorized or inadvertent entry of personnel into high-risk operational areas is one of the primary causes of safety accidents, underscoring the urgent need for effective monitoring and early-warning mechanisms. Furthermore, Fabiano et al. [3] analyzed long-term accident statistics in port environments and demonstrated that personnel intrusion into hazardous operational zones accounts for a considerable proportion of accident causes, making it a critical issue in the safety management of ports and logistics parks. Therefore, developing vision-based automated monitoring technologies to enable intelligent safety warning and reduce accident risks is of significant practical importance.

However, the visual environment of container yards is inherently complex. On the one hand, the operational area spans a wide range of scales, and distant workers or local objects often appear as small targets in images, frequently subject to occlusion by containers, vehicles, or equipment structures. On the other hand, loading and unloading equipment exhibits pronounced pose variations and geometric deformations under different operational states, leading to highly unstable visual appearances. These factors collectively pose substantial challenges to high-precision object detection in container yard scenarios. Experimental studies conducted by Ning et al. [4] in complex railway and industrial environments have shown that small targets, occlusions, and cluttered backgrounds significantly degrade the detection performance of deep learning models.

In recent years, vision-based safety monitoring has evolved from generic object recognition toward task-oriented perception frameworks for complex industrial environments. In the construction domain, Lee et al. [5] developed computer vision-based safety recognition models for worker presence, fall-risk identification, and PPE compliance, demonstrating the practical value of deep learning for onsite safety management. Liu et al. [6] further extended this line of research to a multi-task monitoring framework that jointly considers detection, segmentation, pose estimation, and tracking, highlighting the need for integrated visual perception in dynamic safety-critical scenes. In port and container-terminal scenarios, Cuong et al. [7] proposed a computer-vision-based method for reach-stacker operation assistance by combining object detection and distance estimation, while An et al. [8] improved the robustness of reach-stacker tracking under adverse visual conditions through image enhancement and deep learning. These studies collectively indicate that recent research has shifted from generic detector selection toward task-specific visual safety perception under cluttered backgrounds, small-object conditions, and operational uncertainty.

However, existing methods still show three common limitations when applied to container yard safety monitoring. First, distant workers and local operational targets often appear as small objects, making recall highly sensitive to the quality of shallow features. Second, large handling equipment such as cranes and reach stackers exhibits evident geometric deformation and pose variation, which remain difficult to capture using conventional fixed-grid convolutions. Third, most current vision-based safety monitoring systems emphasize target detection alone, while the semantic interpretation of hazardous operational regions is often weak or absent. As a result, target recognition cannot be naturally translated into region-aware warning decisions. These gaps motivate the development of a fusion framework that combines robust object detection with functional-region segmentation for intelligent safety warning in container yards.

Nevertheless, when applied to container yard environments, existing methods still suffer from several unresolved limitations:

1. Difficulty in small object detection: Distant personnel often occupy only a tiny fraction of the image, and insufficient shallow feature representation leads to low recall rates for small targets.
2. Inadequate modeling of deformable objects: Targets such as cranes exhibit significant geometric deformations across different operational states, which are difficult to capture using conventional convolutions with fixed receptive fields, resulting in inaccurate localization.
3. Limited effectiveness of feature fusion: Traditional bilinear interpolation–based upsampling tends to lose semantic information during multi-scale feature fusion, thereby degrading detection accuracy.
4. Lack of regional semantic awareness: Single-task detection models are unable to determine the safety attributes of the regions where targets are located, making it difficult to support semantic rule–based intelligent safety warning.

To address these challenges, this paper proposes a container yard safety monitoring method based on the YOLOv8 framework, termed FSD-YOLO. In this study, “FSD” denotes the joint integration of functional-region segmentation and deformable object detection. Specifically, the proposed framework combines a functional-region segmentation branch with a deformable object detection branch. In the detection branch, C2fDCN refers to the C2f block enhanced with deformable convolution, which is introduced to improve geometric modeling capability for deformable targets in complex yard environments. The main contributions of this work are summarized as follows:

1. A dual-branch architecture that integrates semantic segmentation and object detection is designed. A SegFormer-based module is employed for pixel-level region segmentation, and decision-level fusion is applied to enable region-aware intelligent safety warning, effectively addressing the lack of regional semantic information.
2. A C2f-shallow module is introduced into the shallow layers of the YOLOv8n backbone to fully exploit fine-grained details in high-resolution features, thereby enhancing small object detection performance.
3. A C2fDCN module is proposed by embedding deformable convolutions into the detection head. By learning adaptive sampling offsets, the receptive field shape is dynamically adjusted, improving the modeling capability for deformable targets such as cranes.
4. The CARAFE content-aware upsampling operator is adopted to replace conventional bilinear interpolation, optimizing the multi-scale feature fusion process through adaptive kernel prediction.
5. A dynamic loss-weighting mechanism for small objects is designed, where bounding box loss weights are adaptively adjusted according to target area, reinforcing the model’s training focus on small targets.

By integrating the above components into the YOLOv8n architecture, extensive experiments conducted on a self-constructed container yard dataset demonstrate that the proposed FSD-YOLO model achieves significant performance improvements. Compared with the baseline YOLOv8n, FSD-YOLO increases mAP50-95 from 0.5394 to 0.6433, corresponding to a relative improvement of approximately 19.3%, while the F1-score improves from 0.8480 to 0.9399, representing an increase of about 10.8%.

The remainder of this paper is organized as follows. Section 2 reviews related work. Section 3 presents the proposed methodology in detail. Section 4 reports experimental results and analysis. Section 5 discusses the findings and their limitations. Finally, Section 6 concludes the paper and outlines directions for future research.

## 2. Related Work

In recent years, deep learning–based object detection techniques have achieved rapid development, providing essential technical support for industrial safety monitoring and perception in complex operational environments. Existing object detection approaches are predominantly built upon general-purpose detection frameworks and can be broadly categorized into two-stage and one-stage methods. Representative two-stage approaches, such as Fast R-CNN, Faster R-CNN, and Mask R-CNN, achieve high detection accuracy through region proposal generation followed by refined classification and regression. However, their multi-stage inference pipelines often incur substantial computational overhead, limiting their applicability in real-time monitoring scenarios [9,10,11]. In contrast, one-stage methods, including the YOLO series, SSD, and RetinaNet, adopt end-to-end detection strategies that significantly improve inference efficiency while maintaining competitive accuracy, making them more suitable for industrial safety monitoring tasks [12,13,14]. In recent years, task-oriented visual safety monitoring studies have further shown that modern industrial safety perception increasingly depends not only on generic detector accuracy but also on robust recognition under occlusion, small-object conditions, and dynamic operational environments [5,6].

In the context of container yards and port safety monitoring, researchers have increasingly applied these object detection methods to the automatic recognition and behavioral analysis of personnel, vehicles, and handling equipment. Xu et al. [15] extracted high-fidelity kinematic information from port surveillance videos to support traffic and operational situation awareness, providing a framework for mapping visual data to physical quantities for risk assessment and early warning. Cuong et al. [7] combined object detection with distance estimation to support remote monitoring and safety decision-making for container yard handling equipment. An et al. [8] addressed the challenge of stable tracking of reach stackers and forklifts under complex illumination and image degradation conditions by integrating image enhancement and deep learning–based tracking strategies, thereby improving the robustness of vehicle trajectory acquisition. Chen et al. [16] further proposed a comprehensive framework for extracting automated guided vehicle (AGV) kinematic information from port videos to enhance traffic safety situation awareness. Kim et al. [17] constructed a multispectral detection dataset for forklifts and personnel in industrial parks and introduced the concepts of hazardous and warning zones, providing valuable references for vision-based alerts against personnel intrusion into dangerous areas. More recent studies have also shown that visual safety monitoring is evolving from simple object recognition toward more integrated perception frameworks that combine detection with scene understanding, risk analysis, and multi-task monitoring [6,7,8].

From a model architecture perspective, one-stage detectors have become the dominant choice for industrial safety monitoring due to their superior inference speed. Existing studies indicate that structural improvements to the YOLO series can further enhance detection performance while preserving real-time capability. For example, several works have incorporated deformable convolution and CARAFE content-aware upsampling modules into the YOLOv8 framework, effectively improving the modeling of deformable targets and small objects [18,19,20,21]. Deformable convolution enhances localization stability by adaptively adjusting sampling positions, thereby better capturing geometric deformations and pose variations. CARAFE improves multi-scale feature fusion by leveraging content-aware feature reassembly mechanisms. Regarding small object detection, survey studies have shown that fully exploiting shallow high-resolution features, enhancing multi-scale feature representations, and introducing loss function optimization strategies during training are effective approaches for improving small object recall and overall detection performance [22]. These observations are highly relevant to container yard scenarios, where distant personnel, cluttered backgrounds, and deformable handling equipment impose stricter requirements on geometric modeling and multi-scale perception.

Beyond object detection, semantic segmentation techniques provide crucial support for region-level modeling in complex operational scenes. SegFormer employs a lightweight Transformer encoder to capture global contextual information, achieving high segmentation accuracy while maintaining efficiency. Mask2Former further proposes a unified mask prediction framework, offering a general modeling paradigm for multi-task segmentation [23,24]. In industrial and port environments, increasing attention has been paid to integrating regional semantic information with object detection results for hazardous area intrusion monitoring and safety assessment. For example, hazardous-area intrusion detection and safety risk visualization studies have shown that explicit region modeling can improve the interpretation of target behavior in safety-critical scenes [25,26]. More recently, region-aware safety perception frameworks combining semantic segmentation with target detection have further demonstrated that semantic-region extraction is an important prerequisite for transforming visual recognition results into warning decisions [27,28]. Therefore, compared with single-task detection models, a joint perception framework that combines object detection and region-level semantic modeling is more suitable for intelligent safety warning in container yard environments.

In summary, existing research has established a solid methodological foundation for container yard safety monitoring in terms of object detection, deformable target modeling, small object enhancement, and semantic segmentation. Nevertheless, real-world container yard scenarios still suffer from several persistent challenges, including insufficient recall for distant small targets, unstable localization of deformable objects such as handling equipment, and inadequate deep fusion between region-level semantic information and object detection results. Motivated by these observations, this paper adopts YOLOv8 as the baseline detection framework and integrates the semantic segmentation capability of SegFormer to propose FSD-YOLO, a fusion-based segmentation and detection method tailored for container yard safety monitoring.

## 3. Proposed Method

**Figure 1:**
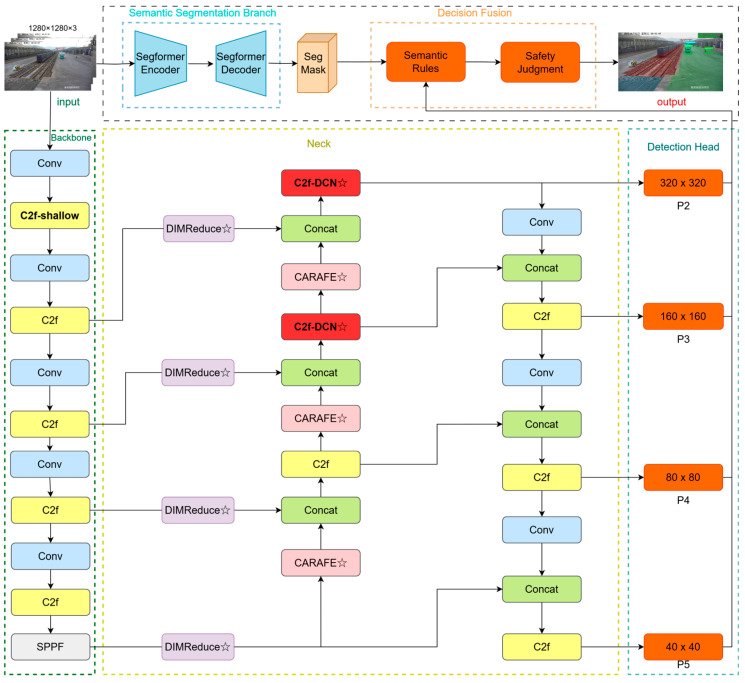
Overall architecture of the proposed FSD-YOLO framework. The star symbols denote the newly introduced/improved modules.

The overall architecture of the proposed FSD-YOLO framework is illustrated in Figure 1. The system adopts a dual-branch fusion design consisting of a semantic segmentation branch, an object detection branch, and a decision-level fusion module. The semantic segmentation branch is built upon SegFormer-B2 to perform pixel-level partitioning of operational regions, which characterizes the spatial distribution of functional areas within the container yard. The object detection branch is developed based on YOLOv8n with multiple structural enhancements, aiming to improve the detection of small-scale and deformable targets. The decision fusion module performs spatial association between segmentation outputs and detection results, and generates safety warnings according to predefined semantic rules. Given an input image, the two branches operate in parallel, and a unified safety judgment is produced at the decision layer.

### 3.1. Overall System Architecture

As shown in Figure 1, the proposed system mainly consists of the following components.

#### 3.1.1. Input Layer

The system takes container yard surveillance images with a resolution of 1280 × 1280 as input. The input image is simultaneously fed into the semantic segmentation branch and the object detection branch, ensuring that region modeling and target perception are conducted under the same visual scale.

#### 3.1.2. Semantic Segmentation Branch

The semantic segmentation branch is located in the upper part of Figure 1 and employs SegFormer-B2 to achieve pixel-level functional-region segmentation [29]. SegFormer adopts a lightweight Transformer encoder to extract multi-scale global representations and a decoder to perform cross-scale feature fusion, achieving a balance between segmentation accuracy and computational efficiency. The segmentation branch outputs multi-class region masks, including background, railway restricted zone, road safe zone, and temporary passage zone, which provide region-level semantic constraints for subsequent safety judgment.

#### 3.1.3. Object Detection Branch

The object detection branch, shown in the lower part of Figure 1, is the primary focus of improvement in this work. The overall detection framework is built upon YOLOv8n, with multiple task-oriented optimizations tailored to container yard scenarios.

- Backbone. The backbone extracts multi-scale features from P1/2 to P5/32. Unlike the original YOLOv8 architecture, a C2f-shallow module is introduced at the P1/2 stage to enhance high-resolution feature representation, thereby improving the modeling of distant small targets. The remaining stages adopt standard convolutional downsampling and C2f blocks, and an SPPF module is employed at the end of the backbone to enlarge the receptive field.
- Neck. The neck adopts a bidirectional feature fusion structure combining FPN and PAN. In the top-down pathway, CARAFE content-aware upsampling is used to replace conventional bilinear interpolation, alleviating semantic information loss during upsampling and improving multi-scale feature fusion quality. In addition, at the P2 and P3 fusion stages, standard C2f blocks are replaced with C2fDCN blocks by introducing deformable convolution, enabling adaptive receptive field adjustment for deformable targets.
- Detection Head. A four-scale detection head is employed, corresponding to feature maps P2/4, P3/8, P4/16, and P5/32. Compared with the original three-scale detection head in YOLOv8, the additional P2 detection layer significantly enhances small-object detection capability, which is particularly suitable for recognizing distant personnel and local operational targets in container yards.

#### 3.1.4. Region Based Safety Assessment Module

The decision fusion module performs spatial association between detection results and segmentation masks. The Region Analysis module determines the functional region in which each detected target is located, while the Safety Rules module generates corresponding safety alerts based on predefined semantic rules.

Through the synergy of these components, FSD-YOLO achieves substantial improvements in detecting small-scale and deformable targets.

### 3.2. Improved Object Detection Model

#### 3.2.1. C2f-Shallow Module

In the original YOLOv8n backbone, feature extraction starts from the P2 layer (stride = 4). Considering that small objects contain richer details in shallow high-resolution feature maps, a C2f-shallow module is added at the P1 layer (stride = 2) to enhance high-resolution feature extraction.

The C2f module integrates the cross-stage partial connection mechanism from CSPNet and the efficient layer aggregation design of ELAN. Given an input feature map $X\in R^{C\times H\times W}$, the computation process can be formulated as:

(1) $$X_{1},X_{2}=\mathrm{Split}({\mathrm{Conv}}_{1\times 1}\left(X)\right)$$

(2) $$Y_{i}=\mathrm{Bottleneck}(Y_{i-1}),\;i=1,2,\ldots ,n$$

(3) $$Y={\mathrm{Conv}}_{1\times 1}(\mathrm{Concat}(X_{1},X_{2},Y_{1},Y_{2},\ldots ,Y_{n}))$$

where Split denotes channel splitting, Bottleneck represents a bottleneck block, and nnn is the number of bottleneck layers.

#### 3.2.2. C2fDCN Module

To enhance modeling capability for deformable targets in complex operational environments, a C2fDCN module is proposed by integrating deformable convolution into the C2f structure. Deformable convolution enables the convolution kernel to adaptively adjust its receptive field by learning spatial offsets, mitigating the limitations of fixed-grid convolution under pose variations and geometric deformation [30].

The deformable convolution operation is formulated as:

(4) $$y(p_{0})=\sum_{p_{n}\in R}w(p_{n})\cdot x(p_{0}+p_{n}+\Delta p_{n})$$

where $p_{0}$ denotes a location on the output feature map, R is the regular sampling grid, $w(p_{n})$ represents convolution weights, and $\Delta p_{n}$ is the learned spatial offset.

**Figure 2:**
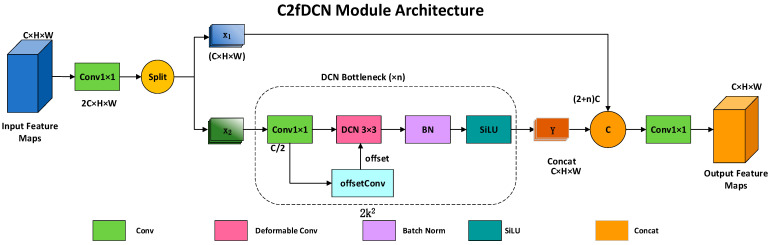
Structure of the proposed C2fDCN module.

As shown in Figure 2, the C2fDCN module preserves the cross-stage partial connection of C2f while introducing deformable convolution for enhanced feature representation. The input feature is first transformed by a 1×1 convolution and split into two branches. One branch directly participates in feature aggregation to retain original information, while the other branch is fed into a DCN Bottleneck submodule. Within the DCN Bottleneck, channel compression, offset prediction, and deformable convolution are sequentially applied, followed by batch normalization and SiLU activation. Multiple DCN Bottleneck units can be stacked to further improve modeling capability. The module output is obtained by concatenating features from all branches and mapping them back to the original channel dimension. In this work, C2fDCN is deployed at the P2 and P3 stages.

#### 3.2.3. CARAFE Upsampling

In multi-scale feature fusion, upsampling operations play a critical role in determining feature reconstruction quality. Conventional bilinear interpolation employs fixed interpolation weights at all spatial locations and lacks the ability to adapt to local semantic content, which often leads to semantic information loss during the upsampling process. To address this issue, the CARAFE [21] module is introduced to replace the conventional upsampling operations in the neck of YOLOv8n, enabling content-aware feature reassembly and improving multi-scale feature fusion performance.

The core idea of CARAFE is to predict position-specific upsampling kernels based on the content information of input features, thereby fully exploiting local contextual semantics when reconstructing high-resolution feature maps. The computation process of CARAFE mainly consists of two stages: upsampling kernel prediction and feature reassembly.

First, given an input feature map X, a content-aware upsampling kernel W is predicted through a mapping function ϕ(⋅), which can be formulated as:

(5) $$W=\phi (X)\in {(R}^{C_{up}\times k^{2}\times H\times W})$$

where k denotes the spatial size of the upsampling kernel, and $C_{up}$ is related to the upsampling factor σ. During the feature reassembly stage, the output value at an arbitrary spatial position (i,j) is obtained by a weighted aggregation of local neighborhood features that are semantically related to this position:

(6) $$Y(i,j)=\sum_{n=-r}^{r}\sum_{m=-r}^{r}W_{n,m}(i,j)\cdot X\left(\lfloor i/\sigma \rfloor +n,\lfloor j/\sigma \rfloor +m\right)$$

where k=2r+1 represents the spatial size of the reassembly kernel.

**Figure 3:**
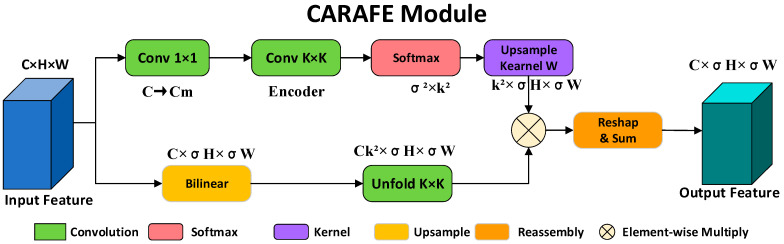
Architecture of the CARAFE content-aware upsampling module.

As illustrated in Figure 3, the CARAFE module consists of two main components: an upsampling kernel prediction path and a feature reassembly path. In the kernel prediction path, the input feature is first compressed using a 1×1 convolution to reduce computational cost. Then, a k×k convolution is applied to encode local contextual information from the compressed features. Afterward, a Softmax normalization is performed to generate position-specific upsampling kernel weights, ensuring spatial normalization and stability of the predicted reassembly kernels.

In the feature reassembly path, the input feature map is first upsampled by a factor of σ using bilinear interpolation to obtain a base high-resolution feature map. Subsequently, an Unfold operation is applied to extract a k×k neighborhood for each spatial position. These neighborhood features are then multiplied element-wise with the predicted upsampling kernels. Finally, through reshaping and summation operations, the weighted neighborhood features are aggregated into a single output value, producing an upsampled feature map of size C×σH×σW.

Compared with conventional bilinear interpolation, CARAFE leverages content-aware kernel prediction and feature reassembly mechanisms to more effectively preserve local semantic consistency and fine-grained structural information during multi-scale feature fusion. This property is particularly beneficial for the stable propagation of small-object features. Therefore, in this work, CARAFE is uniformly adopted to perform upsampling operations in the neck of YOLOv8n, enhancing feature representation capability in complex container yard scenarios.

#### 3.2.4. Dynamic Loss Weighting for Small Objects

In large-scale operational scenarios such as container yards, distant personnel and local targets usually occupy only a very small number of pixels in images. Consequently, their regression errors contribute little to the overall loss, causing the model to bias toward optimizing medium- and large-scale targets during training, which degrades small-object detection performance. To alleviate this issue, this study proposes a dynamic loss-weighting mechanism based on target area, which adaptively adjusts the weight of bounding-box regression loss during training to assign greater emphasis to small objects [31].

The pixel area of a target bounding box is defined as:

(7) A = w × h

where w and h denote the width and height of the bounding box, respectively. According to the statistical distribution of target scales in the dataset, an area threshold is set as $A_{th}=24\times 24=576$ pixels. A gain factor γ (set to 1.5 in this work) is then introduced to define the instance-level dynamic weighting coefficient:

(8) $$\alpha =\left\{\begin{array}{ll} \gamma & \mathrm{if}\;A\;<{\;A}_{\mathrm{th}}\\ 1.0 & \mathrm{otherwise} \end{array}\right.$$

To further prevent training instability caused by excessive weighting of individual small targets, a batch-level small-object ratio adjustment strategy is incorporated. Specifically, for each training batch, the proportion of small objects $r_{small}$ is calculated, and the overall bounding-box regression loss weight is adaptively adjusted as:

(9) $$\lambda_{\mathrm{box}}^{\mathrm{dynamic}}=\lambda_{\mathrm{box}}^{\mathrm{base}}\times (1+(\gamma -1)\times r_{\mathrm{small}})$$

where $\lambda_{\mathrm{box}}^{\mathrm{base}}$ denotes the base weight of the bounding-box regression loss.

**Figure 4:**
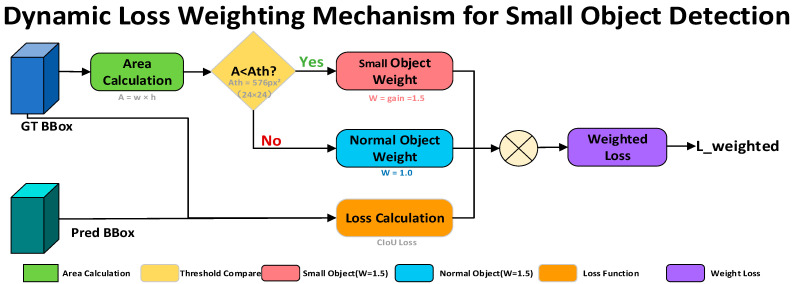
Illustration of the dynamic loss-weighting mechanism for small objects.

As shown in Figure 4, the overall workflow of the proposed dynamic loss-weighting mechanism is illustrated. During training, the target area is first computed from the annotated bounding boxes to determine the target scale category. According to the predefined area threshold, small targets are assigned higher loss weights, while normal targets retain their original weights. The predicted bounding boxes and ground-truth boxes jointly participate in the CIoU loss computation, which is then reweighted using the proposed dynamic weighting strategy before backpropagation. By moderately amplifying the regression errors associated with small objects, the model focuses more on small-object localization accuracy during parameter updates.

The proposed dynamic loss-weighting mechanism exhibits two notable advantages. First, the area-based weighting strategy is simple to implement and incurs negligible computational overhead, allowing seamless integration into existing detection frameworks. Second, by incorporating batch-level adjustment based on the proportion of small objects, the method effectively mitigates gradient oscillation issues that may arise from excessive weighting of individual targets. In complex container yard scenarios, this mechanism enables stable enhancement of the model’s learning capability for distant small objects, providing effective support for improving overall small-object detection performance.

### 3.3. Semantic Segmentation Model

To obtain pixel-level semantic information of different functional regions within container yards, SegFormer-B2 is adopted as the semantic segmentation model. In the proposed framework, the segmentation branch is introduced not merely to improve standalone region segmentation accuracy, but to provide stable functional-region priors for the downstream decision-level safety warning module. Because the predicted region labels are directly involved in target-region assignment and subsequent warning generation, the segmentation model must not only preserve the boundaries between adjacent functional regions but also maintain moderate computational cost in the dual-branch, real-time framework. Therefore, the choice of SegFormer-B2 should be understood as a task-driven design decision rather than an arbitrary replaceable backbone selection. SegFormer is an efficient Transformer-based semantic segmentation architecture composed of a hierarchical Transformer encoder and a lightweight all-MLP decoder, which supports this design objective well.

#### 3.3.1. Model Architecture

The encoder of SegFormer follows a four-stage hierarchical design, where each stage consists of multiple Transformer blocks to progressively extract multi-scale semantic features from local to global levels. Unlike standard Vision Transformers that employ non-overlapping patch partitioning, SegFormer introduces overlapped patch embedding, which enhances continuity modeling between adjacent regions and better preserves edge information and local structural details. In addition, an efficient self-attention mechanism is adopted in the encoder to reduce computational complexity, enabling the model to maintain high efficiency under high-resolution inputs. The SegFormer-B2 encoder contains approximately 25 M parameters and is pretrained on the ADE20K dataset, providing a strong initialization for semantic segmentation in complex scenes.

The decoder adopts an all-MLP structure without introducing additional complex convolutional or attention modules. Multi-scale features from different encoder stages are first aligned to the same spatial resolution through upsampling operations, then concatenated along the channel dimension, and finally fused via a linear projection. This process can be formulated as:

(10) $$F={\mathrm{Linear}}_{C_{\mathrm{embed}}}\left(\mathrm{Concat}\left(\mathrm{Upsample}(F_{i})\right)\right),\;i=1,2,3,4$$

The fused feature is subsequently mapped to pixel-level prediction results through a linear classification layer:

(11) $$M={\mathrm{Linear}}_{N_{\mathrm{cls}}}(F)\;$$

where $F_{i}$ denotes the output feature of the i-th encoder stage, $C_{embed}$ represents the channel dimension after feature fusion, and $N_{cls}$ is the number of segmentation classes.

#### 3.3.2. Segmentation Task Definition

According to the actual operational environment of rail-road intermodal container yards, a semantic segmentation task with four functional region classes is constructed to characterize the safety attributes of different areas. Specifically, the segmentation categories include background, railway restricted zone, road safe zone, and temporary passage zone.

The background corresponds to non-functional areas. The railway restricted zone covers railway tracks and their adjacent high-risk operational regions. The road safe zone mainly refers to vehicle passage roads where personnel are allowed to move normally. The temporary passage zone indicates areas where personnel are allowed to pass during specific operational stages but should remain cautious. Through this region taxonomy, the model provides explicit spatial semantic constraints for subsequent safety judgment.

#### 3.3.3. Training Configuration

The semantic segmentation model is fine-tuned based on the pretrained weights NVIDIA/segformer-b2-finetuned-ade-512-512. The experimental dataset contains 1305 images, which are split into training, validation, and test sets with a 7:2:1 ratio. The input resolution is fixed at 512 × 512 pixels. During training, the AdamW [32] optimizer is employed with an initial learning rate of $6\times 10^{-5}$, together with a polynomial learning rate decay strategy. The batch size is set to 8, and the model is trained for 50 epochs. A 500-step learning rate warm-up is introduced to enhance training stability. To further accelerate training and reduce GPU memory consumption, FP16 mixed-precision training is adopted.

To clarify the rationale for choosing SegFormer-B2, we further compared multiple SegFormer variants under the same training setting. The results show that SegFormer-B4 achieves the highest mIoU (0.9650) but also introduces the largest model complexity and the slowest inference. SegFormer-B3 provides only marginal accuracy improvement over B2, while B0 and B1 are lighter but slightly inferior in functional-region modeling accuracy. SegFormer-B2 achieves 0.9638 mIoU with 27.35 M parameters and 21.64 ms latency, remaining very close to the larger variants in segmentation accuracy while maintaining substantially lower computational cost. Therefore, SegFormer-B2 was retained in this study because it provides a more appropriate balance between functional-region segmentation quality and system efficiency for region-aware safety warning in container yards.

During training, the segmentation performance on the validation set improves consistently. The final mIoU increases from an initial 88.1% to 96.39%, and the pixel accuracy reaches 98.57%. These results demonstrate that the adopted SegFormer-B2 model can accurately delineate the spatial boundaries of different functional regions in complex container yard scenarios, providing reliable region-level semantic information support for subsequent detection and safety decision modules.

### 3.4. Decision Fusion Module

The decision fusion module is designed to perform spatial association between object detection results and semantic segmentation outputs, and to generate safety state assessment and warning outputs under region-level semantic constraints. By modeling the spatial relationship between detected targets and functional regions, this module maps low-level visual perception results into safety judgments with clear engineering significance.

#### 3.4.1. Region Assignment Algorithm

For each detected target iii, the corresponding bounding box is represented as:

(12) $$B_{i}=(x_{1},y_{1},x_{2},y_{2})$$

First, the region corresponding to this bounding box is extracted from the semantic segmentation mask MMM as the region of interest (ROI):

(13) $${ROI}_{i}=M[y_{1}:y_{2},x_{1}:x_{2}]$$

Subsequently, the number of pixels belonging to each semantic category within the ROI is counted to characterize the spatial overlap between the target and different functional regions. For each category c∈{0,1,2,3}, the pixel count is defined as:

(14) $${count}_{c}=\sum_{(x,y)\in {ROI}_{i}}I\left(label(x,y)=c\right)$$

where I(⋅) denotes the indicator function.

Based on these statistics, the maximum-overlap principle is adopted to determine the primary functional region to which the target belongs. The assigned region label is given by:

(15) $${region}_{i}=\arg\underset{c}{\max}\;{count}_{c}$$

Meanwhile, to quantify the strength of association between the target and the assigned region, the pixel proportion of the dominant region within the target ROI is further computed as:

(16) $${ratio}_{i}=\frac{{count}_{{region}_{i}}}{\sum_{c}{count}_{c}}$$

This ratio can serve as a basis for further extensions such as risk-level refinement or confidence evaluation.

#### 3.4.2. Safety Warning Rules

After determining the region assignment for each target, the system generates the corresponding safety state label according to the functional region type in which the target is located. Following commonly used formulations of risk classification functions, the safety decision rule is formalized as a piecewise function:

(17) $$\mathrm{Risk}(i)=\left\{\begin{array}{ll} \mathrm{DANGER} & \mathrm{if}\;{\mathrm{region}}_{i}=1\\ \mathrm{CAUTION} & \mathrm{if}\;{\mathrm{region}}_{i}=3\\ \mathrm{SAFE} & \mathrm{if}\;{\mathrm{region}}_{i}=2\\ \mathrm{NORMAL} & \mathrm{if}\;{\mathrm{region}}_{i}=0 \end{array}\right.$$

Here, category 1 corresponds to the railway restricted zone, which represents a high-risk operational area; once a target is detected within this region, a danger alert is immediately triggered. Category 3 denotes the temporary passage zone, where a caution warning is issued to remind personnel to remain vigilant. Category 2 represents the road safe zone, indicating that the target is in an allowed passage area. Category 0 corresponds to the background region, for which no safety alert is generated.

Through the proposed decision fusion mechanism, object detection results are effectively integrated with region-level semantic information, enabling a semantic elevation from “whether a target exists” to “whether a target is in a hazardous state.” This provides a clear and interpretable decision basis for real-time safety monitoring and warning in container yard scenarios.

## 4. Experimental Results and Analysis

To verify the effectiveness of the proposed FSD-YOLO, experiments are conducted on a self-constructed container yard safety monitoring dataset. This section first introduces the dataset and evaluation metrics, followed by a description of the experimental settings.

### 4.1. Dataset and Evaluation Metrics

#### 4.1.1. Dataset

**Figure 5:**
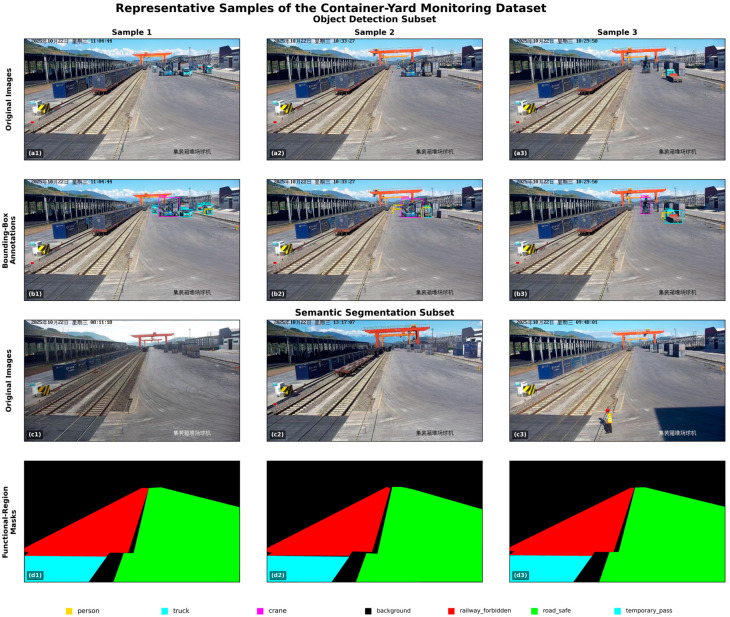
Representative samples from the self-constructed container-yard monitoring dataset: (**a**): (**a1**–**a3**) original images from the object detection subset; (**b**): (**b1**–**b3**) corresponding bounding box annotations for person, truck, and crane; (**c**): (**c1**–**c3**) original images from the semantic segmentation subset; (**d**): (**d1**–**d3**) corresponding functional-region masks, including background, railway forbidden, road safe, and temporary pass.

To evaluate the applicability of the proposed framework in realistic yard operations, we constructed a container-yard safety monitoring dataset from surveillance videos collected in a rail-road intermodal logistics park. The acquisition device is a spherical surveillance camera (iDS-2DF8425ILXR-FW/S2, Hangzhou Hikvision Digital Technology Co., Ltd., Hangzhou, China), and the viewing angle changes irregularly over time. The sampled videos were selected from two weeks of working periods and cover different lighting conditions and weather conditions. After video screening and split construction, the data were organized as a self-constructed dataset. Figure 5 presents representative samples from the dataset. The upper two rows show the original images and corresponding bounding-box annotations for the object-detection subset, illustrating typical yard targets such as persons, trucks, and cranes under realistic surveillance viewpoints. The lower two rows show the original images and corresponding functional-region masks for the semantic-segmentation subset, where the restricted operational region, safe traffic region, and temporary passage region are annotated at the pixel level.

The original video screening process produced 4300 candidate frames. However, not all extracted frames contained annotated instances of the three target categories considered in this study. Since the object detection experiments were constructed using images with valid YOLO-format bounding-box annotations, frames without target annotations were excluded from the final split. As a result, the effective object detection dataset used in the experiments contains 2988 labeled images, including 2091 training images, 597 validation images, and 300 test images. A total of 5780 annotated objects are included, comprising 3822 persons, 1497 trucks, and 461 cranes. In terms of target scale, the dataset is strongly dominated by small objects, including 4993 small targets, 755 medium targets, and 32 large targets.

The semantic segmentation dataset contains 1305 image-mask pairs for pixel-level annotation of functional regions in the container yard. The pixel-level category ratios are 42.61% for background, 16.28% for the restricted operational region, 33.86% for the safe traffic region, and 7.25% for the temporary passage region.

#### 4.1.2. Evaluation Metrics

For the object detection task, multiple widely used evaluation metrics are employed to comprehensively assess model performance. The mean Average Precision (mAP50-95) is adopted as the primary metric to evaluate overall detection performance across different IoU thresholds ranging from 0.5 to 0.95. In addition, mAP50 is reported to reflect detection capability under a relatively loose overlap criterion. Furthermore, Precision, Recall, and F1-score are used to assess the trade-off between false positives and false negatives.

For the semantic segmentation task, the mean Intersection over Union (mIoU) is used as the core evaluation metric to measure segmentation accuracy across different functional regions. Pixel accuracy is also reported to indicate the overall correctness of region classification. Through comprehensive analysis of these metrics, the performance of FSD-YOLO in both detection accuracy and region-level semantic modeling can be systematically evaluated.

### 4.2. Experimental Settings

All experiments are conducted within the PyTorch 2.0 framework for model construction, training, and evaluation. The experimental environment is equipped with an Intel Core i9 processor, an NVIDIA RTX 4090 GPU with 24 GB of memory, and 64 GB of system RAM. In addition to the training environment, a dedicated runtime benchmark was conducted to quantitatively evaluate the inference efficiency of the detection branch, the segmentation branch, and the complete end-to-end fusion framework on the target hardware. For object detection model training, the AdamW optimizer is used for parameter optimization. The initial learning rate is set to 0.001 and gradually decayed to 0.01 using a cosine annealing strategy. The batch size is set to 10, and all input images are resized to 1280 × 1280. The model is trained for 150 epochs. To enhance model generalization, various data augmentation strategies are applied during training, including Mosaic augmentation, random horizontal flipping, and HSV color space perturbation.

For semantic segmentation, the model is fine-tuned from the pretrained weights NVIDIA/segformer-b2-finetuned-ade-512-512. The AdamW optimizer is also adopted, with an initial learning rate of $6\times 10^{-5}$. The batch size is set to 8, and the input image resolution is fixed at 512 × 512. The model is trained for 50 epochs. With appropriate training configurations and pretrained weight initialization, the segmentation model converges stably within a relatively short training period and achieves high segmentation accuracy.

### 4.3. Experimental Analysis

#### 4.3.1. Training Process Analysis

**Figure 6:**
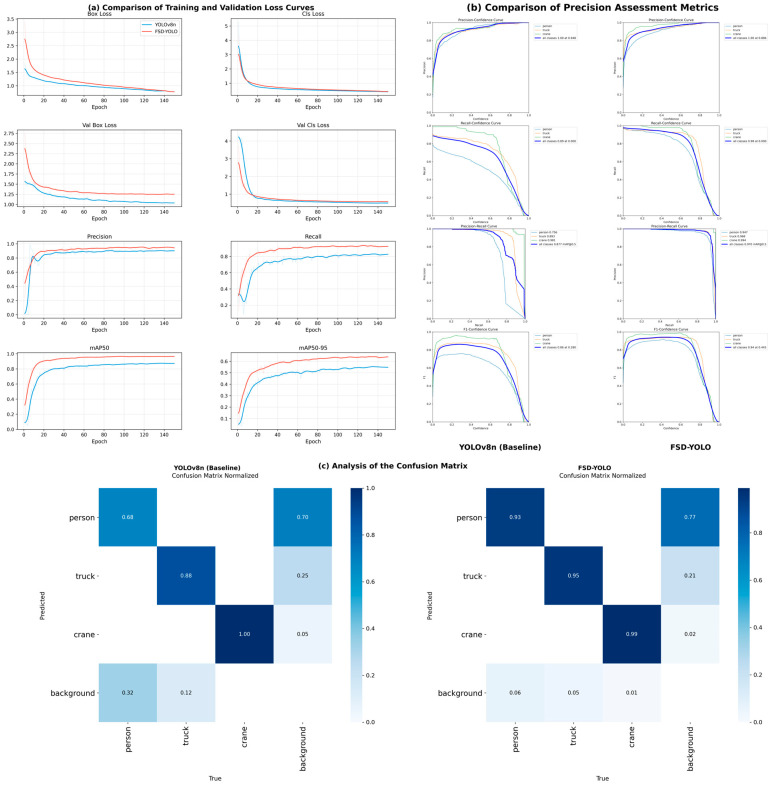
Performance comparison between FSD-YOLO and YOLOv8n on the container yard dataset: (**a**) loss convergence curves during training and validation; (**b**) comparison of evaluation metrics under different confidence thresholds; (**c**) normalized confusion matrices of the two models.

To systematically evaluate the effectiveness of the proposed FSD-YOLO for container yard safety monitoring, a comparative study is conducted against the baseline YOLOv8n under the same experimental settings. Figure 6 presents the loss convergence behaviors, detection performance curves during training, and the confusion matrix results for the two models.

From the training and validation loss curves, it can be observed that FSD-YOLO achieves faster convergence and lower final loss values for both bounding-box regression and classification. Compared with the baseline model, the box loss of FSD-YOLO decreases more rapidly in the early training stage and stabilizes at a lower level in the later stage, indicating that introducing the C2fDCN module and CARAFE upsampling significantly improves localization accuracy in complex scenes. Meanwhile, the classification loss curve of the improved model exhibits smaller fluctuations throughout training, suggesting more stable feature representation, which benefits discrimination among multiple object categories.

In terms of detection performance, FSD-YOLO outperforms the baseline in both precision and recall. In the late training stage, FSD-YOLO reaches a precision of 0.9534 and a recall of 0.9267, which are substantially higher than those of YOLOv8n (0.9327 and 0.7775, respectively). This indicates a notable reduction in missed detections, especially for small objects and deformable targets. The mAP curves further confirm this trend: the mAP50 of FSD-YOLO exceeds 0.90 after approximately the 50th epoch and finally stabilizes at 0.9565; the mAP50-95 increases to 0.6433, showing a significant improvement over the baseline (0.5394). These results demonstrate that the proposed enhancements consistently improve detection performance under different IoU thresholds.

To further analyze performance under varying confidence thresholds, Figure 6b compares the precision–confidence, recall–confidence, PR, and F1–confidence curves. It can be observed that across the entire confidence range, the precision and recall curves of FSD-YOLO remain above those of the baseline, indicating better robustness. The area under the PR curve is noticeably larger, and the AP50 values for all three categories exceed 0.95. The F1–confidence curve shows that FSD-YOLO achieves the best F1 score of 0.9399 at a confidence threshold of approximately 0.4, suggesting an improved balance between precision and recall.

The confusion matrix results are shown in Figure 6c. Compared with YOLOv8n, FSD-YOLO exhibits significantly higher values along the diagonal, indicating improved classification accuracy across categories. Specifically, the recognition accuracies for person, truck, and crane reach 92.7%, 95.4%, and 91.2%, respectively, while the false positives on background are reduced. This suggests that incorporating region semantics and enhancing feature modeling effectively improves category discrimination in complex backgrounds.

Overall, FSD-YOLO demonstrates clear advantages over the baseline in training stability, convergence speed, and final detection performance. These results validate the effectiveness of the proposed modules in complex container yard scenarios and provide reliable technical support for vision-based real-time safety monitoring and warning applications.

#### 4.3.2. Error Analysis

To better understand the remaining limitations of the proposed detector, we further analyzed false positives and false negatives on the 300-image test set. The analysis shows that 74 images contain at least one detection error, with 59 false positives and 41 false negatives in total. Most errors are concentrated in the person category, which accounts for 49 false positives and 28 false negatives. In terms of target scale, the majority of the errors are associated with small objects: 58 of the 59 false positives and 38 of the 41 false negatives belong to the small-object category. These results indicate that distant and small personnel targets remain the most challenging cases in complex container-yard scenes.

**Figure 7:**
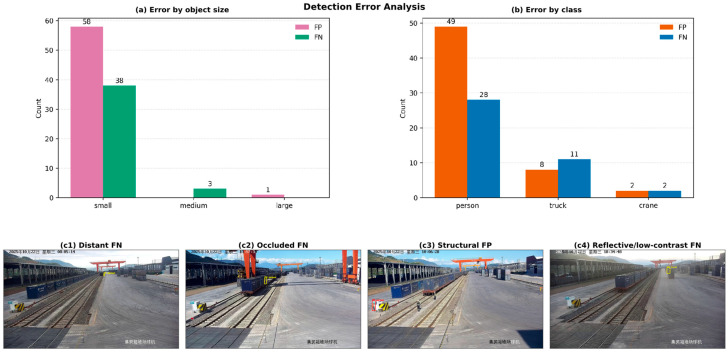
Error analysis of FSD-YOLO on the container yard test set: (**a**) error distribution by object size; (**b**) error distribution by class; (**c**): (**c1**) distant false negative; (**c2**) occluded false negative; (**c3**) structural false positive; (**c4**) reflective or low-contrast false negative. The yellow boxes denote false negatives (FN), i.e., ground truth targets that were not detected, and the red boxes denote false positives (FP), i.e., incorrect detections.

Figure 7 further visualizes the statistical distribution of the errors and several representative failure cases. As shown in Figure 7a,b, the residual errors are highly concentrated on small objects and the person category. Figure 7(c1,c2) presents two typical false-negative cases, showing that distant or partially occluded persons are still difficult to detect reliably. Figure 7(c3) illustrates a structural false-positive case, where background regions with person-like local patterns are incorrectly recognized as targets. Figure 7(c4) shows a reflective or low-contrast false-negative example, indicating that adverse illumination conditions may further weaken the discriminability of distant small targets. Overall, the error analysis suggests that the main remaining challenge of the proposed detector lies in the stable perception of small, distant, and visually ambiguous targets under complex yard backgrounds.

#### 4.3.3. Real-Time Performance Analysis

To quantitatively validate the real-time capability of the proposed framework, we further benchmarked the inference efficiency of the object detection branch, the semantic segmentation branch, and the complete end-to-end fusion framework on the target hardware. All measurements were conducted on an NVIDIA RTX 4090 GPU with batch size 1 and FP16 inference after a warm-up stage. The detailed runtime results are summarized in Table 1.

As shown in Table 1, the object detection branch achieves an average latency of 7.91 ms, a P50 latency of 7.91 ms, a P95 latency of 7.97 ms, and a throughput of 126.44 FPS, with a peak GPU memory usage of 492.1 MB. The semantic segmentation branch achieves an average latency of 21.64 ms, a P50 latency of 21.64 ms, a P95 latency of 22.39 ms, and a throughput of 46.21 FPS, while consuming 411.4 MB of peak GPU memory. For the complete end-to-end dual-branch fusion framework, the average latency is 31.36 ms, the P50 latency is 31.34 ms, the P95 latency is 31.81 ms, and the achieved throughput is 31.89 FPS, with a peak GPU memory usage of 483.7 MB.

These results indicate that the proposed framework maintains practical real-time capability even after integrating the functional-region segmentation branch. In other words, the introduced semantic-region modeling module enhances region-aware safety reasoning without sacrificing the deployability of the overall system on modern industrial GPU hardware.

### 4.4. Comparison with Advanced Methods

To further verify the overall performance of the proposed method in complex container yard scenarios, FSD-YOLO is compared with multiple mainstream object detection models, including the YOLOv8 series, YOLOv11 series, and representative two-stage detectors. The comparison results under the same dataset and unified evaluation metrics are summarized in Table 2.

From the perspective of detection accuracy, FSD-YOLO achieves the best or near-best performance across the core metrics. In particular, mAP50-95 reaches 0.6433, representing a 19.3% improvement over the baseline YOLOv8n. Moreover, it still surpasses the largest YOLOv8x by 14.1%, indicating that the proposed multi-module enhancements significantly improve localization accuracy and robustness under stricter IoU thresholds. For mAP50, FSD-YOLO achieves 0.9565, which is higher than YOLOv8l by 7.3%, demonstrating a clear advantage even under a looser overlap criterion.

Regarding the overall balance between precision and recall, FSD-YOLO achieves Precision = 0.9534, Recall = 0.9267, and F1 = 0.9399, outperforming both YOLOv8 and YOLOv11 variants. These results indicate that the proposed method reduces false positives while effectively lowering the miss rate, leading to a better precision–recall trade-off in complex backgrounds and small-object-dense scenarios.

From the perspective of real-time performance, Table 2 also shows that FSD-YOLO maintains competitive inference efficiency while significantly improving detection accuracy. Its average latency is 7.91 ms, corresponding to 126.44 FPS, which is substantially faster than the representative two-stage detectors, whose throughput is only about 35.81–36.41 FPS. Although FSD-YOLO is slower than the smallest one-stage baselines such as YOLOv8n and YOLOv8s, it provides a much stronger accuracy level while still satisfying practical real-time requirements. This demonstrates that the proposed design achieves a more favorable trade-off between accuracy and efficiency for safety monitoring in container yards.

Compared with two-stage detectors, FSD-YOLO also shows a clear advantage in mAP50-95. Although Faster R-CNN, Fast R-CNN, and Mask R-CNN achieve relatively competitive mAP50, their overall performance under stricter IoU thresholds remains inferior to FSD-YOLO. In addition, these two-stage methods exhibit substantially higher computational complexity (GFLOPs > 800), making them less suitable for real-time safety monitoring. By contrast, FSD-YOLO provides strong detection accuracy while maintaining practical deployability, making it more suitable for real-time container yard safety monitoring tasks.

Furthermore, to validate applicability under resource-constrained environments, a lightweight variant, FSD-YOLO-Lite, is developed. With only 3.01 M parameters, FSD-YOLO-Lite achieves mAP50-95 = 0.6269 and F1 = 0.9170, improving upon YOLOv8n by 16.2% in mAP50-95 while running at 167.01 FPS. These results demonstrate that, through channel compression and structural simplification, FSD-YOLO-Lite preserves most of the performance gains of the full model while offering a more efficient solution for edge devices and embedded deployment.

### 4.5. Ablation Study

To systematically evaluate the independent contribution of each proposed module and their synergy, ablation experiments are conducted on the self-constructed container yard dataset using YOLOv8n as the baseline. The results are summarized in Table 3, where the shallow C2f module, C2fDCN module, CARAFE upsampling, and the dynamic loss weighting mechanism (DLW) [33] are gradually introduced, and different combinations are compared.

First, after introducing the shallow C2f-shallow module (M1), mAP50-95 increases from 0.5394 to 0.6285, yielding a 16.5% improvement. Meanwhile, precision and recall become 0.8891 and 0.9262, respectively. This indicates that enhancing cross-stage feature interaction at high-resolution feature layers effectively reduces missed detections of distant personnel and small targets caused by insufficient details, confirming the importance of shallow feature enhancement for small-object detection.

Second, introducing C2fDCN alone (M2) increases mAP50-95 to 0.6158, a 14.1% improvement over the baseline. With a relatively high precision (0.8919), recall increases to 0.9105, suggesting that deformable convolution improves localization stability for targets with substantial geometric deformation and pose variation (e.g., cranes) by adaptively adjusting sampling locations.

For feature fusion, applying CARAFE upsampling (M3) results in mAP50-95 = 0.6258, improving upon the baseline by 16.0%. CARAFE achieves a high precision of 0.9438, indicating that content-aware upsampling better preserves semantic information during multi-scale feature reassembly and mitigates semantic drift during fusion.

The dynamic loss weighting mechanism (M4) also yields positive gains. After introducing DLW, mAP50-95 increases to 0.6274, with precision and recall reaching 0.9283 and 0.9182, respectively. This suggests that assigning higher loss weights to small objects during training helps the model learn discriminative patterns for small targets more effectively, producing a stable overall performance improvement.

Further analysis of module combinations shows that pairwise combinations of C2fDCN, CARAFE, and DLW can improve detection performance to some extent, but the gains are smaller than introducing shallow C2f alone. This implies that without high-resolution shallow feature support, structural modeling or feature reassembly alone may not fully unleash their potential.

When all four modules are incorporated to form the complete model FSD-YOLO (M5), the best performance is achieved, with mAP50-95 = 0.6433, corresponding to a 19.3% improvement over the baseline, while precision, recall, and F1 score also show clear advantages. This indicates a strong synergy among shallow feature enhancement, deformable modeling, content-aware feature fusion, and dynamic optimization for small objects, enabling simultaneous improvement in small-object detection and localization accuracy for complex targets.

In addition, the lightweight FSD-YOLO-Lite achieves mAP50-95 = 0.6269 with significantly reduced complexity, providing performance close to the full model and further demonstrating good generalization under different computational constraints.

**Figure 8:**
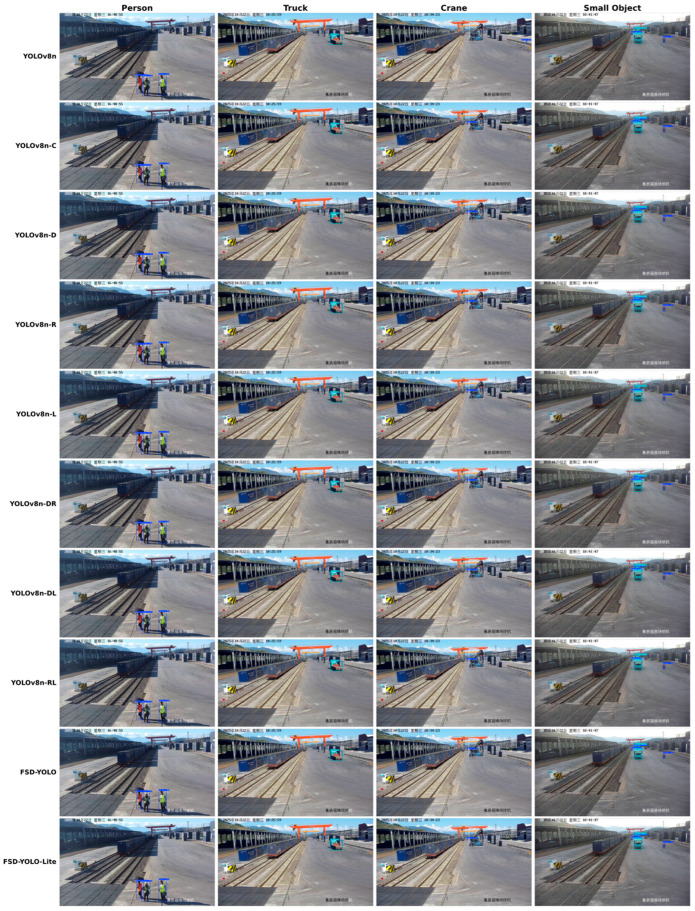
Qualitative comparison of detection results across different methods in representative container yard scenarios. These colored frames indicate the detection results of the object detection branch. Blue boxes denote ‘person’, cyan boxes denote ‘truck’, and white boxes denote ‘crane’.

Figure 8 visualizes detection results in typical container yard scenes, covering representative targets such as persons, trucks, cranes, and distant small objects. The baseline YOLOv8n exhibits frequent missed detections for distant persons and small targets, with some targets not detected or detected with low confidence scores. Under occlusion or complex backgrounds, bounding boxes also show noticeable localization shifts. After introducing the shallow C2f module, detection completeness for distant persons improves, with more stable and consistent bounding boxes, demonstrating the benefit of high-resolution feature enhancement.

After further incorporating C2fDCN, detection performance on deformable targets (e.g., cranes) improves significantly. Bounding boxes better fit target outlines under varying poses and structural deformations, and localization shifts are reduced. In dense multi-target scenes, the baseline and some intermediate variants still suffer from missed or duplicate detections, whereas FSD-YOLO can more accurately distinguish adjacent targets with more coherent overall results. Under occlusion, FSD-YOLO maintains stable predictions based on visible cues, achieving higher bounding-box completeness and confidence than other methods. Overall, FSD-YOLO demonstrates stronger robustness and higher detection reliability in complex yard environments. Although FSD-YOLO-Lite shows slightly reduced stability under extreme occlusion, it still performs significantly better than the baseline.

**Figure 9:**
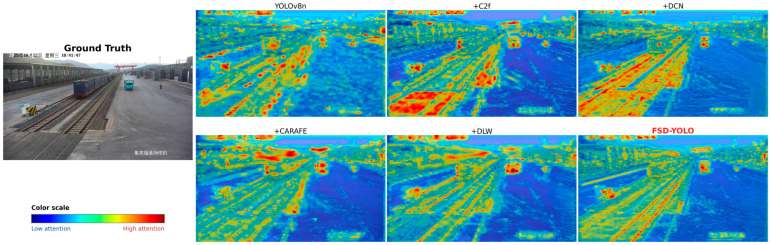
XGrad-CAM visualizations of feature activation maps for different variants in the ablation study.

Figure 9 presents XGrad-CAM [34] visualization results to analyze model attention regions during detection decisions. For the baseline YOLOv8n, high-response regions are scattered, and some background areas are incorrectly activated, indicating limited discriminative capability under complex scenes. After introducing shallow C2f, model attention becomes stronger on targets such as persons and vehicles, but background interference remains.

With the inclusion of C2fDCN and CARAFE, high-response regions gradually concentrate on target bodies and key structural parts. In particular, around deformable targets such as trucks, feature responses become more continuous and clearer. In FSD-YOLO, high-response regions align closely with true target locations, while background activation is markedly suppressed, demonstrating that the synergy of multiple modules improves feature discriminability and spatial focus. These observations provide explainability evidence supporting the effectiveness of FSD-YOLO in complex container yard scenarios.

### 4.6. Training and Results of the Semantic Segmentation Model

**Figure 10:**
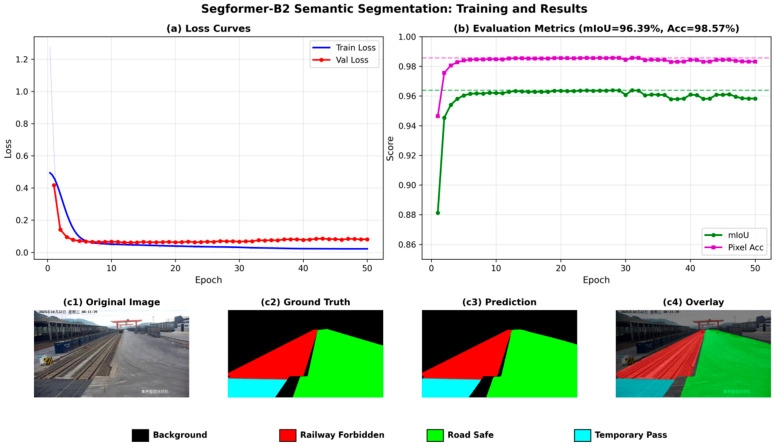
Training curves and segmentation results of the SegFormer-B2 model: (**a**) loss curves; (**b**) evaluation metric curves; (**c**): (**c1**–**c4**) visualization of segmentation results, including the original image, ground truth, predicted mask, and overlay visualization.

Figure 10 illustrates the training process and qualitative segmentation results of the SegFormer-B2–based semantic segmentation model on the container yard dataset, which are used to evaluate the convergence behavior, quantitative performance, and practical segmentation effectiveness for functional region modeling.

From the training process shown in Figure 10a, the training loss decreases rapidly within the first few epochs, converging from an initial value of approximately 1.28 to around 0.06, while the validation loss follows a similar trend and stabilizes at approximately 0.068. No obvious divergence between the training and validation losses is observed, indicating good generalization capability and training stability of the model.

Figure 10b presents the variation in segmentation evaluation metrics over training epochs. The mIoU steadily increases from an initial 88.1% and stabilizes at 96.39% in the later training stage, while the pixel accuracy improves from 94.6% to 98.57%. Both metrics reach high levels within a relatively small number of training epochs, demonstrating that SegFormer-B2 can effectively learn discriminative features of different functional regions in container yard scenes and achieve strong performance in both overall region partitioning accuracy and pixel-level consistency.

To further visually assess segmentation quality, Figure 10(c1–c4) shows the segmentation results of a representative sample, including the original image, manually annotated ground truth, model prediction, and overlay visualization of the predicted mask on the original image. In these visualizations, black denotes the background region, red indicates the railway restricted zone, green represents the road safe zone, and cyan corresponds to the temporary passage zone. As can be observed, the model accurately distinguishes railway operational areas from regions accessible to personnel and vehicles, with clear and continuous boundaries and high intra-region consistency.

Under complex scene conditions, the model still exhibits strong robustness. Even in the presence of illumination changes, large-scale variations, or partial occlusions, SegFormer-B2 can reasonably infer functional regions based on contextual semantic information, avoiding obvious region misclassification or boundary fragmentation. These results indicate that the segmentation model can provide stable and reliable region-level semantic constraints for subsequent object detection results, serving as high-quality prior information for rule-based safety decision-making.

### 4.7. Performance of the Fusion System

**Figure 11:**
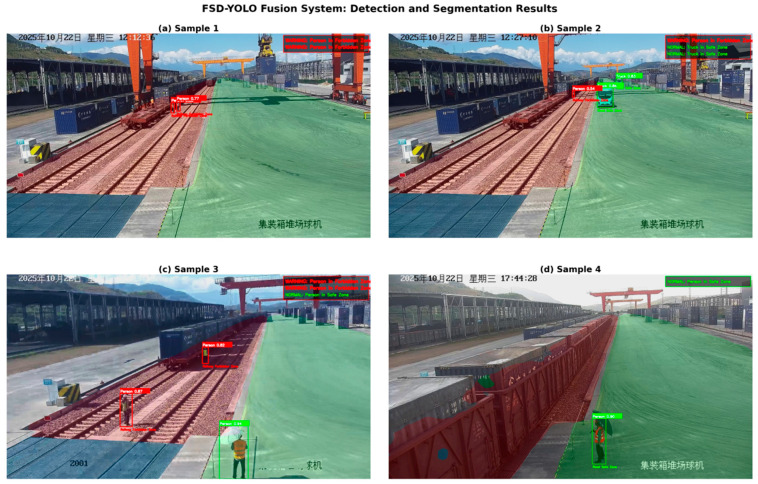
Operational results of the FSD-YOLO fusion system in real container yard scenarios. Green boxes/green text indicate that the target is in a safe area, while red boxes/red text indicate that the target is in a dangerous area. The upper-right label provides the corresponding safe/dangerous status prompt for the targets shown in the figure.

Figure 11 presents the operational results of the FSD-YOLO fusion system in real rail-road intermodal container yard scenarios, comprehensively visualizing object detection outputs, semantic segmentation results, and region-aware safety warning responses. The four selected examples cover different operational distances, personnel distributions, and region combinations, providing a representative overview of system performance in practical applications.

From the object detection results, the system can stably detect persons, trucks, and cranes under complex background conditions. Even for distant, small-scale targets or partially occluded objects, the predicted bounding boxes adequately cover target entities while maintaining high confidence scores. This demonstrates that the proposed detection branch exhibits strong robustness in small-object perception and complex structural modeling, providing reliable object-level information for subsequent safety assessment.

When combined with semantic segmentation results, different functional regions are clearly delineated in the spatial domain. The railway restricted zone, road safe zone, and temporary passage zone are distinctly separated in the overlay visualization, closely matching the actual structural layout of the container yard. Even under illumination variations or camera viewpoint changes, the segmentation results remain continuous and consistent, indicating that the segmentation branch provides stable and trustworthy region-level semantic constraints for detected targets.

Based on these outputs, the decision fusion module performs spatial association between detected targets and their corresponding region types and automatically generates safety warnings according to predefined rules. As shown in Figure 11, when a person enters the railway restricted zone, the system immediately triggers a danger alert and highlights the target in red; when a target is located in a temporary passage zone, a caution warning is issued; and when personnel or vehicles are within the road safe zone, they are labeled as being in a safe state. These results demonstrate that the fusion system not only performs object recognition but also effectively establishes the association among targets, regions, and risk levels, enabling meaningful safety-aware perception.

## 5. Discussion

The experimental results show that the proposed FSD-YOLO framework achieves a practical balance between detection accuracy, functional-region perception, and deployment efficiency for container yard safety monitoring. By integrating semantic segmentation with object detection, the framework enables region-aware warning generation in railway restricted zones, road safe zones, and temporary passage zones. In addition, although larger segmentation backbones such as SegFormer-B3 and B4 can produce slightly higher mIoU, the improvement over B2 is limited compared with the increase in model complexity and inference latency. Therefore, SegFormer-B2 remains a more balanced choice for the proposed dual-branch real-time warning framework.

At the same time, the current runtime benchmark confirms that the complete fusion framework can satisfy practical real-time requirements on modern industrial GPU hardware. However, the error analysis indicates that the remaining detection failures are still dominated by small and distant targets, especially distant personnel and partially occluded instances. Moreover, the current dataset is collected from a single logistics park, so the generalization ability of the framework to other yards with different layouts, equipment configurations, weather conditions, and camera viewpoints still requires further verification. In addition, the warning rules are currently designed based on engineering knowledge and operational requirements, but formal expert validation has not yet been completed.

Future work will focus on expanding the dataset to more logistics hubs and more diverse environmental conditions, further improving the perception of small and distant targets, and conducting systematic expert-oriented validation of the warning rules. In addition, integrating the proposed framework with other visual monitoring or multimodal sensing techniques may further improve the robustness and practical applicability of region-aware safety warning in complex industrial environments.

## 6. Conclusions

This study proposes FSD-YOLO, a fusion-based safety perception framework for rail-road intermodal container yards that integrates semantic segmentation and object detection to enable region-aware intelligent warning. The framework combines a SegFormer-based functional-region segmentation branch with an improved YOLOv8-based detection branch, so that target-level detection results can be interpreted under region-level semantic constraints.

Experimental results on the self-constructed real-world container yard dataset verify the effectiveness of the proposed method. The segmentation branch achieves an mIoU of 96.39%, providing reliable semantic support for region assignment and risk assessment. In the detection branch, the introduction of the shallow C2f module, C2fDCN, CARAFE, and dynamic loss weighting significantly improves detection performance, and the final FSD-YOLO achieves an mAP50-95 of 0.6433 and an F1 score of 0.9399, corresponding to improvements of 19.3% and 10.8%, respectively, over YOLOv8n.

In addition, the proposed framework demonstrates practical deployment potential for real-time safety monitoring in container yard operations. A lightweight variant, FSD-YOLO-Lite, is further developed for resource-constrained scenarios and still maintains competitive accuracy. Overall, the results indicate that FSD-YOLO provides an effective and practical solution for vision-based safety monitoring and intelligent warning in complex industrial environments.

## Figures and Tables

**Table 1:** Runtime benchmark of the detection branch, segmentation branch, and complete fusion framework on NVIDIA RTX 4090 (batch size = 1, FP16 inference).

Component	Avg Latency (ms)	P50 (ms)	P95 (ms)	FPS	Peak VRAM (MB)
Detection branch	7.91	7.91	7.97	126.44	492.1
Segmentation branch	21.64	21.64	22.39	46.21	411.4
End-to-end fusion framework	31.36	31.34	31.81	31.89	483.7

**Table 2:** Comparison with YOLOv8/YOLOv11 series and two-stage detectors on the container yard dataset.

Model	Parameters (M)	GFLOPs	mAP50-95	mAP50	P	R	F1	Avg Latency (ms)	FPS
YOLOv8n	3.01	4.1	0.5394	0.8435	0.9327	0.7775	0.8480	4.46	224.34
YOLOv8s	11.14	14.3	0.5664	0.8691	0.9230	0.8197	0.8683	4.80	208.46
YOLOv8m	25.86	39.5	0.5626	0.8742	0.9600	0.7731	0.8565	6.89	145.06
YOLOv8l	43.63	82.7	0.5716	0.8917	0.9014	0.8336	0.8662	9.02	110.87
YOLOv8x	68.16	129.1	0.5640	0.8737	0.9205	0.8088	0.8610	11.53	86.76
YOLOv11n	2.59	3.2	0.5154	0.8184	0.9276	0.7326	0.8186	5.18	193.12
YOLOv11s	9.43	10.8	0.5431	0.8705	0.9191	0.8046	0.8580	5.26	190.21
YOLOv11m	20.06	34.1	0.5643	0.8559	0.8965	0.7988	0.8448	6.48	154.38
Fast R-CNN R50	41.7	828.0	0.5771	0.9335	0.9335	0.6369	0.7572	27.47	36.41
Mask R-CNN R50	44.2	1040.0	0.5781	0.9340	0.9340	0.6405	0.7599	27.82	35.94
Faster R-CNN R50	41.7	828.0	0.5777	0.9434	0.9434	0.6412	0.7635	27.93	35.81
FSD-YOLO (Detection)	7.59	177.2	0.6433	0.9565	0.9534	0.9267	0.9399	7.91	126.44
FSD-YOLO-Lite (Detection)	3.01	58.5	0.6269	0.9480	0.9260	0.9083	0.9170	5.99	167.01

**Table 3:** Ablation results on the container yard dataset. ‘✓’ indicates that the corresponding module is included in the model configuration.

Method	C2f	C2fDCN	CARAFE	DLW	mAP50	mAP50-95	Precision	Recall
Baseline (M0)					0.8435	0.5394	0.9327	0.7775
+C2f-shallow (M1)	✓				0.9424	0.6285	0.8891	0.9262
+C2fDCN (M2)		✓			0.9343	0.6158	0.8919	0.9105
+CARAFE (M3)			✓		0.9336	0.6258	0.9438	0.8893
+DLW (M4)				✓	0.9497	0.6274	0.9283	0.9182
+C2fDCN+CARAFE		✓	✓		0.9305	0.6136	0.9194	0.9160
+C2fDCN+DLW		✓		✓	0.9348	0.6158	0.8954	0.8993
+CARAFE+DLW			✓	✓	0.9407	0.6143	0.9126	0.9197
FSD-YOLO (M5)	✓	✓	✓	✓	0.9565	0.6433	0.9534	0.9267
FSD-YOLO-Lite	✓	✓	✓	✓	0.9480	0.6269	0.9260	0.9083

## Data Availability

Data supporting the findings of this study are available from the corresponding author upon reasonable request.
